# Inhibition of neutrophil activity improves cardiac function after cardiopulmonary bypass

**DOI:** 10.1186/1476-9255-4-21

**Published:** 2007-10-10

**Authors:** Ulf Abdel-Rahman, Stefan Margraf, Tayfun Aybek, Tim Lögters, José Bitu-Moreno, Ieda Francischetti, Tilmann Kranert, Frank Grünwald, Joachim Windolf, Anton Moritz, Martin Scholz

**Affiliations:** 1 Department of Thoracic and Cardiovascular Surgery, Johann Wolfgang Goethe University, Frankfurt am Main, Germany; 2 Department of Traumatology and Hand Surgery, Heinrich-Heine University, Düsseldorf, Germany; 3 Department of Vascular Surgery, Faculdade Medicina Marilia (FAMEMA), Marilia, Brasil; 4 Department of Nuclear Medicine, Johann Wolfgang Goethe University, Frankfurt am Main, Germany

## Abstract

**Background:**

The arterial in line application of the leukocyte inhibition module (LIM) in the cardiopulmonary bypass (CPB) limits overshooting leukocyte activity during cardiac surgery. We studied in a porcine model whether LIM may have beneficial effects on cardiac function after CPB.

**Methods:**

German landrace pigs underwent CPB (60 min myocardial ischemia; 30 min reperfusion) without (group I; n = 6) or with LIM (group II; n = 6). The cardiac indices (CI) and cardiac function were analyzed pre and post CPB with a Swan-Ganz catheter and the cardiac function analyzer. Neutrophil labeling with technetium, scintigraphy, and histological analyses were done to track activated neutrophils within the organs.

**Results:**

LIM prevented CPB-associated increase of neutrophil counts in peripheral blood. In group I, the CI significantly declined post CPB (post: 3.26 ± 0.31; pre: 4.05 ± 0.45 l/min/m^2^; p < 0.01). In group II, the CI was only slightly reduced (post: 3.86 ± 0.49; pre 4.21 ± 1.32 l/min/m^2^; p = 0.23). Post CPB, the intergroup difference showed significantly higher CI values in the LIM group (p < 0.05) which was in conjunction with higher pre-load independent endsystolic pressure volume relationship (ESPVR) values (group I: 1.57 ± 0.18; group II: 1.93 ± 0.16; p < 0.001). Moreover, the systemic vascular resistance and pulmonary vascular resistance were lower in the LIM group. LIM appeared to accelerate the sequestration of hyperactivated neutrophils in the spleen and to reduce neutrophil infiltration of heart and lung.

**Conclusion:**

Our data provides strong evidence that LIM improves perioperative hemodynamics and cardiac function after CPB by limiting neutrophil activity and inducing accelerated sequestration of neutrophils in the spleen.

## Background

Cardiac surgery using cardiopulmonary bypass (CPB) is associated with impaired cardiac function at the end of surgery [1,2]. However, the underlying pathophysiological mechanisms are multifold and unsolved yet. Among other pathogenic factors the increase in unspecific innate immune responses seems to play a central role in CPB-related pathogenicity. It is known that CPB and ischemia/reperfusion are related to postoperative sequelae due to aberrant neutrophil activation and inflammatory responses [3-5]. This unspecific immune activation is reminiscent of the systemic immune response syndrome (SIRS) and may be elicited by the contact of patient blood with artificial surfaces of the extracorporeal circuits [1,2]. Activated neutrophils are known to mediate endothelial dysfunction via secretion of proteolytic enzymes such as elastase or oxygen radicals, followed by edema, tissue destruction [3,4], and impairment of hemodynamics [6]. In addition to these systemic effects, activated neutrophils may particularly damage the ischemic heart and lung during the reperfusion phase after opening of the aortic crossclamp [7]. Neutrophils contribute to vascular resistance and to microvascular blood flow by having to squeeze through capillaries and forming a temporary obstruction. During ischemia (and CBP) the pressure that keeps these cells moving is lost and they appear to become adherent. When flow is restored they contribute to the "no-reflow" phenomenon and exacerbate damage [8-15].

Many efforts have been done in the past to limit the CPB-related inflammatory sequelae. However, strategies such as leukocyte filtration in the arterial line of the heart-lung machine were of limited success [16,17]. Recently, we reported on the effects of a novel leukocyte inhibition module (LIM) in a porcine model [18]. LIM catalyzes physiological cellular mechanisms that are important for the stabilization of the innate immune system. Upon neutrophil contact with the biofunctional LIM-matrix consisting of open porous polyurethane foam as a carrier of stably immobilized anti-Fas (anti-CD95) monoclonal antibodies, rapid inactivation occurs via Fas-signaling. To date, the major paradigm of Fas-signaling has been the induction of apoptosis and the subsequent engulfment of preapoptotic neutrophils [19,20]. However, we were able to show earlier, that stimulation of Fas on neutrophils may also lead to apoptosis-independent inactivation within minutes after contact with FasL or with respective agonists [21].

In our recently published experiments [18] we showed that LIM rapidly inactivated neutrophil function and prevented overshooting immune responses due to CPB. For example, the proinflammatory cytokine TNF-alpha was significantly reduced in blood samples over time. Moreover, the tissue damage markers CK and CK-MB were found to be reduced when animals were operated with CPB and LIM [18]. We assumed that hyperactivated neutrophils perioperatively may participate in the impairment of cardiac function, a phenomenon that has been related to the pathogenic features of CPB [1,2]. Therefore, we proposed that inhibition of neutrophil function by LIM may stabilize cardiac function.

Here, we report on our data showing the effects of LIM on CPB-related decrease of cardiac function in a porcine model.

## Methods

### Porcine model and cardiopulmonary bypass

The investigation conforms to the Guide for the Care and Use of Laboratory Animals published by the US National Institutes of Health (NIH Publication NO. 85-23, revised 1996). The study was done after ethical consideration and approval by the regional government.

Pigs (German landrace; 50.75 +/-1.18 kg) were allocated to two groups (each n = 6). All pigs were sham-operated (median sternotomy) with CPB, without (group I; 62 ± 6 min myocardial ischemia and 30 ± 2 min reperfusion) or with (group II; 63 ± 7 min myocardial ischemia and 30 ± 2 min reperfusion) LIM. Anesthesia was maintained consistently with sufentanyl, pancuronium and propofol. Ventilation was performed with a FiO_2 _of 0.5 and a pCO_2 _of 35–40 mmHg. After anticoagulation by systemic administration of 300 IU/kg heparin (Liquemin™; Roche, Grenzach-Wyhlen, Germany), CPB was instituted with a Quadrox™ capillary membrane oxygenator and tubing set including an arterial filter (Pall, 40 μm, Dreieich, Germany; group I), or in addition the leukocyte inhibition module (LIM, Leukocare, Munich, Germany; group II). LIM consists of a thermoplastic housing with a volume of 160 ml. An open porous polyurethane foam carries immobilized agonistic IgM anti-Fas antibodies (clone CH11; Coulter-Immunotech, Hamburg, Germany). The circuit was primed with 1500 ml Ringer's lactate, 500 ml 6% hydroxyethyl starch (HES), 100 ml 20% mannitol, and 150 U/kg of heparin using a prebypass filter (Pall, 0.2 μm). Additional heparin was administered, when activated clotting time (ACT) fell below 400 s. A flow of 2.4 l/min/m^2 ^body surface was applied. The left ventricle was vented through the cardioplegic needle in the ascending aorta. Aortic crossclamp time and reperfusion time were 60 and 30 minutes, respectively in all pigs. Antegrade cold blood cardioplegia was used (arresting dose: 1000 ml) and reinfused (400 ml) every 20 min. After 30 minutes of reperfusion animals were weaned from CPB. Heparin was fully antagonized with protamine sulphate at the end of CPB. One hour after end of CPB pigs were euthanized.

### Blood sampling

Blood samples were obtained immediately before onset of CPB and 10 minutes after weaning from CPB. Blood gas and leukocyte counts were routinely determined with a blood gas analyzer, Cell-Dyn 3500R (Abbott, Wiesbaden, Germany).

### Cardiac function analysis

Hemodynamic parameters were measured in steady state conditions, before CPB and 15 min after weaning from CPB.

### Cardiac index

Left ventricular performance was evaluated with the conductance catheter technique (Leycom CFA-512, Leyden, Holland) by determination of the end systolic pressure volume relationship (ESPVR), end diastolic pressure volume relationship (EDPVR). Pulmonary vascular resistance index (PVRi), systemic vascular resistance index (SVRi), and cardiac index (CI), were assessed as parameters for myocardial pressure relationships. All indexed parameters were normalized for body surface area (m^2^).

Cardiac output was determined by duplicate injection at 4°C (10 ml) into the Swan-Ganz catheter in parallel by the conductance catheter in the left ventricular cavity. The conductance catheter was calibrated according to the results measured by the thermo dilution method.

Systemic vascular resistance index (SVRi) was determined by using the following equation: SVRi = (MAP – CVP)/CO/body surface area (dyn.sec/cm^5^/m^2^) where CVP is central venous pressure. Pulmonary vascular resistance index (PVRi) was calculated accordingly: PVRi = (PAP-LAP)/CO/body surface area (dyn.sec/cm^5^/m^2^) where PAP is mean pulmonary artery pressure.

#### Conductance Catheter Technique

After placement of the conductance catheter to the left ventricular cavity a 20 kHz, 4 mA current is applied on the 12 catheter electrodes, which divide the ventricle into 6 segments. The electric field generated by the current applied allows measurement of the electric conductance within each segment. Differing voltage within a pair of electrodes is inverse proportional to segmental volume. Ventricular volume is calculated using the following equation:

V(t) = ∑_i _= 1–5 V_i_(t) = 1/α)(L^2^/σ) [G_i_(t)-G_i_p]

V (t) left ventricular volume

α correction factor

L distance of electrodes

σ specific conductance of blood

G(t) left ventricular conductance

G(p) parallel conductance

A pressure tip transducer in the conductance catheter measures left ventricular pressure. Pressure volume loop relation is plotted in a pressure volume diagram and a pressure volume loop array of curves is yielded in varying preload using a clamp for inferior vena cava (IVC) occlusion. The slope of end systolic pressure volume points result in the end systolic pressure volume relationship (ESPVR) and describes myocardial contractility. Similarly, the slope of the end diastolic pressure volume points yields the end diastolic pressure volume relationship (EDPVR), and documents myocardial elastance.

### ELISA

Serum samples were obtained from porcine blood and stored at -20°C. Commercial ELISAs were used to determine serum levels of TNF-α (Becton Dickinson, Heidelberg, Germany), CK, and CK-MB (Roche Mannheim, Germany).

### Neutrophil labeling and scintigraphy

Radioactive labelling and scintigraphy was carried out in the Department of Nuclear Medicine, Johann Wolfgang Goethe University Frankfurt after approval by the local commission on radiological protection. The labeling procedure has been done according to the guidelines of the German society of Nuclear Medicine (maximum activity of 740 MBq) and adaptation of the consensus protocol for the porcine blood [22]. Briefly, fresh full arterial blood (120 ml) was obtained from the animal for neutrophil isolation. Neutrophils were isolated from 80 ml blood by 60 min. gravitational sedimentation in citrate buffer (17% ACD-A) and 17% HES (10%) followed by centrifugation of the carefully removed supernatant at 150 g for 5 min. Cell pellet was harvested and resuspended in 1 ml autologous plasma. Plasma was prepared from 40 ml full blood by centrifugation in 17% ACD-A at 2000 g for 10 min. Isolated neutrophils were labeled with 1 ml 99mTc-Exametazime (HMPAO) for 10 min. at room temperature. 3 ml autologous plasma were added and sample was centrifuged at 150 g for 5 min. Subsequently, the supernatant was carefully separated from the cell pellet and stored for the analysis of cell-free radioactivity. Pellet was washed with 4 ml plasma and cells were again resuspended in 15 ml plasma. The efficacy of the labelling procedure was calculated as cell-bound radioactivity × 100/total activity used for labelling. Labelled cells were re-transfused into the animal at onset of CPB. After euthanizing and washing out the blood from the vasculature the total body distribution of the radioactivity was analyzed with scintigraphy for 30 min.

### Histology and staining procedures

Tissue samples were fixed in 4% formaldehyde and embedded in paraffin according to standard procedures. Sections (5 μm) were stained with hematoxylin-eosin for microscopic examination. In addition, chloroacetate esterase staining was performed for specific detection of neutrophils.

### Electron microscopy

Tissue samples were processed for ultrastructural analysis as described previously [23]. Briefly, tissue was fixed with 2.5% glutaraldehyde, postfixed in 1% osmium tetroxide, dehydrated in ethanol, and embedded in resin (Durcupan-Epon; Fluka Chemie GmbH, Buchs, Germany). Thin sections were contrasted with uranyl acetate and lead citrate, and viewed with a microscope (model JEM 2000 CX; JEOL, Arishima, Japan).

### Statistical analysis

Statistical analysis was carried out using the StatView (version 5.0) for Windows software (SAS Institute, Inc, Cary, NC) for repeated assessment of hemodynamic parameters. Wilcoxon test was used to calculate significancies between groups. Differences were considered significant at a probablility level less than 0.05. Data are presented as mean ± standard deviation of mean.

## Results

### Effects of LIM on leukocyte counts

LIM has been shown earlier to prevent the increase in leukocyte numbers and to reduce the functional neutrophil activity [18,24]. In order to correlate LIM-related effects on hemodynamics and cardiac function, leukocyte numbers were measured pre- and post CPB. As expected, an increase of leukocyte numbers has been measured in the control group but not in the LIM group (Table 1). This increase was largely due to the increase of neutrophil numbers but not of lymphocyte numbers. As functional proinflammatory and tissue damage parameter, TNF-α and CK/CK-MB, respectively were found to be lower in the LIM group (Table 1).

**Table 1:** Perioperative inflammatory and tissue damage markers

	Pre-CPB		Post-CPB	
	
	Control	LIM	Control	LIM
Neutrophils (×10^3^/μl)	5.9 ± 0.8	6.4 ± 0.3	13.4 ± 2.3	7.2 ± 1.8
PBL (×10^3^/μl)	7.5 ± 2.1	7.9 ± 1.1	8.8 ± 0.4	8.2 ± 0.9
TNF-α (pg/ml)	68.4 ± 38.9	89.0 ± 25.3	255.3 ± 64.1	112.4 ± 55.7
CK (U/l)	418.1 ± 39.3	397.6 ± 22.0	727.9 ± 75.7	645.8 ± 89.4
CK-MB (U/l)	339.8 ± 44.7	384.9 ± 77.3	592.6 ± 79.3	517.5 ± 69.6

CK: creatine kinase; CPB: cardiopulmonary bypass; PBL: peripheral blood lymphocytes; TNF: tumor necrosis factor

### Effects of LIM on cardiac function

The cardiac function has been analyzed by the thermodilution and conduction catheter technique.

**Figure 1:**
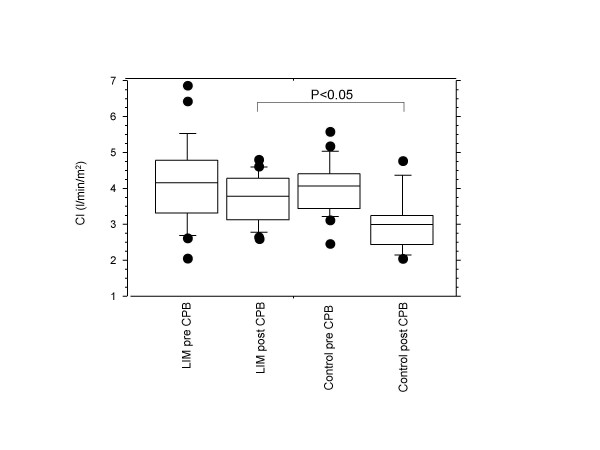
Boxplot depiction of Cardiac index values obtained for the control group and for the LIM group, pre- and postoperatively. In the control group but not in the LIM group, the difference between pre- and post CPB values was statistically significant (p < 0.01). The post-CPB intergroup difference was also statistically significant (p < 0.05).

As shown in Figure 1, the cardiac indices in group I were significantly reduced after CPB (pre CBP: 4.05 ± 0.67 l/min/m^2^; post CPB: 3.26 ± 0.56 l/min/m^2^, p < 0.01). In group II, the cardiac indices were found to be only slightly decreased post CPB, however the difference between pre and post CPB was not significant (pre CPB: 4.21 ± 1.14 l/min/m^2^; post CPB: 3.86 ± 0.71 l/min/m^2^, p = 0.23). The intergroup difference for CI data post CPB (group I: 3.26 ± 0.56 l/min/m^2^; group II: 3.86 ± 0.71 l/min/m^2^) was statistically significant (p < 0.05).

**Figure 2:**
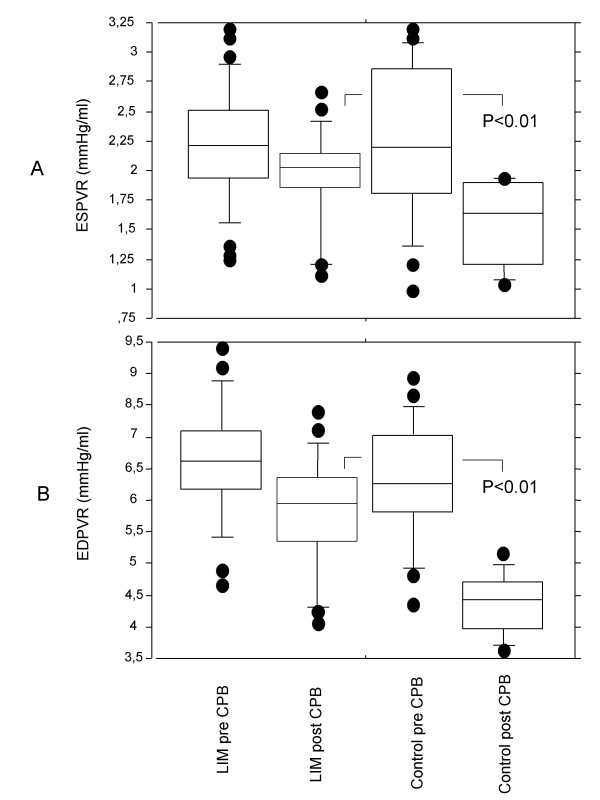
Boxplot depiction of pre-load independent (A) end systolic pressure volume relationship (ESPVR) and (B) end diastolic pressure volume relationship (EDPVR) obtained for the control group and for the LIM group, pre- and postoperatively. In the control group but not in the LIM group, the differences between pre- and post CPB values for ESPVR and EDPVR were statistically significant (p < 0.001). The post-CPB intergroup differences were also statistically significant (p < 0.01).

To explain the LIM-mediated stabilization of CI values, the slopes of end systolic pressure volume relationship (ESPVR) and end diastolic pressure volume relationship (EDPVR) as parameters for myocardial contractility and elastance, respectively, were measured (Figure 2). Data for ESPVR (Figure 2A) in group I were significantly lower after CPB (pre CPB 2.32 ± 0.63 mmHg/ml; post CPB: 1.57 ± 0.42 mmHg/ml, p < 0.001). In the LIM group no significant decrease of ESPVR was found (pre CPB: 2.19 ± 0.49 mmHg/ml; post CPB: 1.93 ± 0.4 mmHg/ml, p = 0.06). Similar data were found for EDPVR values (Figure 2B) with stabilized EDPVR values in the LIM group. EDPVR values in group I were found to be significantly decreased post CPB (pre CPB: 6.19 ± 1.53 mmHg/ml; post CPB: 4.15 ± 0.78 mmHg/ml, p < 0.001). For group II the slight decrease (pre CPB: 6.75 ± 1.5 mmHg/ml; post CPB: 5.92 ± 1.04 mmHg/ml) was not significant (p = 0.38). Intergroup differences for both ESPVR and EDPVR were significant (p < 0.01).

**Figure 3:**
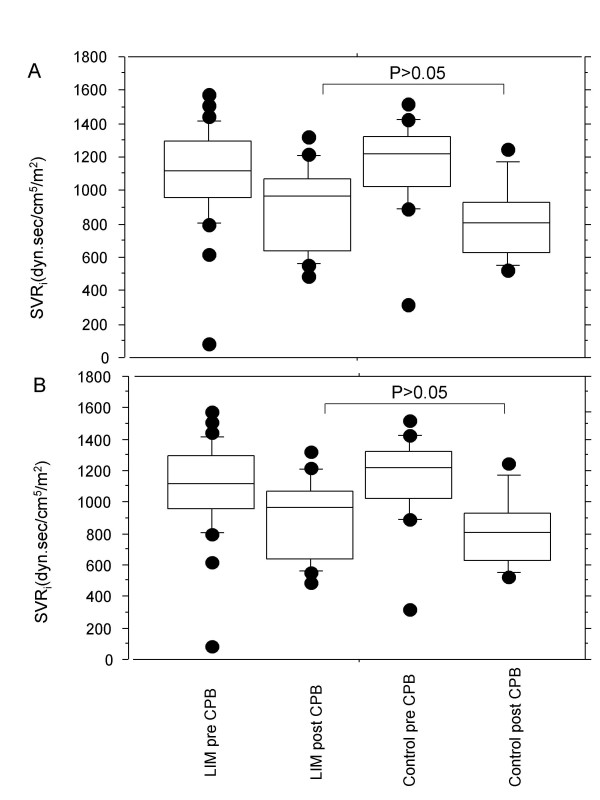
Boxplot depiction of hemodynamic parameters (A) systemic vascular resistance index (SVRi) and (B) pulmonary vascular resistance index (PVRi) obtained for the control group and for the LIM group, pre- and postoperatively. Post CPB intergroup differences for PVRi but not for SVRi were statistically significant (p < 0.01).

In order to evaluate a possible beneficial effect of LIM on systemic and pulmonary hemodynamics, the systemic vascular resistance index (SVRi) and the pulmonary vascular resistance index (PVRi) were measured (Figure 3). Figure 3A depicts the values of the SVRi (n = 6). Post CPB, SVRi values were slightly lower (pre CPB: 1210 ± 128 dyn.sec/cm^5^/m^2^; post CPB: 795 ± 114 dyn.sec/cm^5^/m^2^) compared with pre-operative values in both groups. However, there was no significant intergroup difference. In contrast, the PVRi values increased up to 3-fold post operatively in group I (pre CPB: 190 ± 72 dyn.sec/cm^5^/m^2^; post CPB: 375 ± 134 dyn.sec/cm^5^/m^2^) but not in the LIM group. Post operative PVRi values in the LIM group remained at baseline level (Figure 3B). The post CPB intergroup difference was statistically significant (p < 0.01).

### Cardiac and pulmonary tissue infiltration

**Figure 4:**
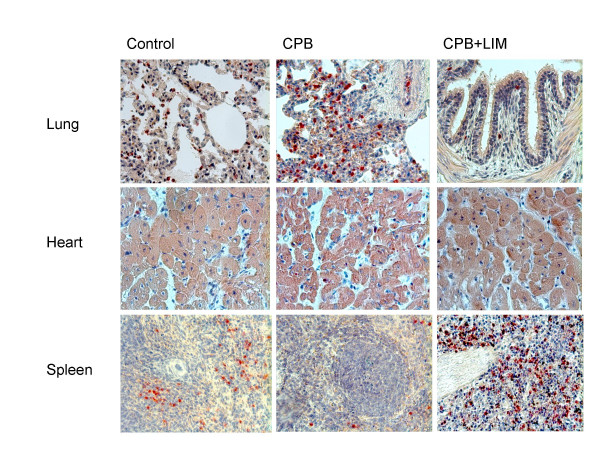
Chloroacetate esterase staining of heart and lung paraffin sections. Representative tissue samples for untreated healthy animals, animals undergoing CPB, and animals undergoing CPB with LIM. Magnification is 200-fold.

To study the possibility whether LIM may exert its beneficial effects on hemodynamics and cardiac function by reducing neutrophil tissue infiltration, tissue sections of heart and lung were stained with neutrophil specific chloracetate-esterase (Figure 4). Semi quantitative evaluation of tissue sections from CPB-treated pigs revealed neutrophil tissue infiltration in heart and lung when compared with sections from untreated control pigs. In tissue sections from LIM-treated pigs reduced numbers of neutrophils in heart and lung were found compared with the CPB group. High numbers of neutrophils were detected in the spleen of LIM-treated pigs but not in control pigs.

**Figure 5:**
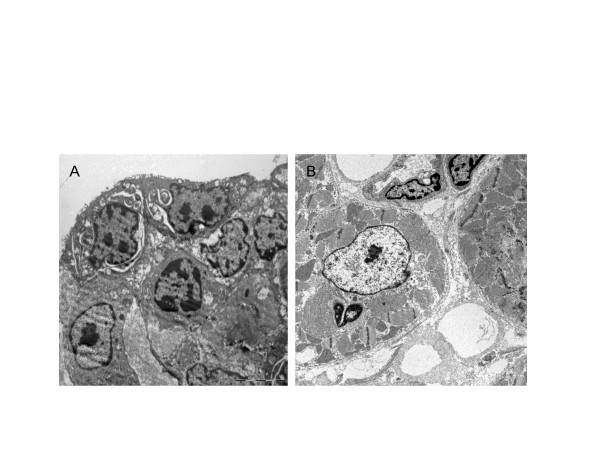
Electron microscopic microphotographs of accumulated neutrophils within the epicardium (A) and within the left ventricular heart muscle (B) after CPB.

Electron microscopy qualitatively confirmed that CPB-mediated neutrophil activation may lead to an accumulation of PMN in the epicardium and to direct interactions between neutrophils and heart muscle cells within the left ventricular myocardium (Figure 5). In tissue samples from LIM-treated animals neutrophils could not be detected within the myocardium.

### Scintigraphy

**Figure 6:**
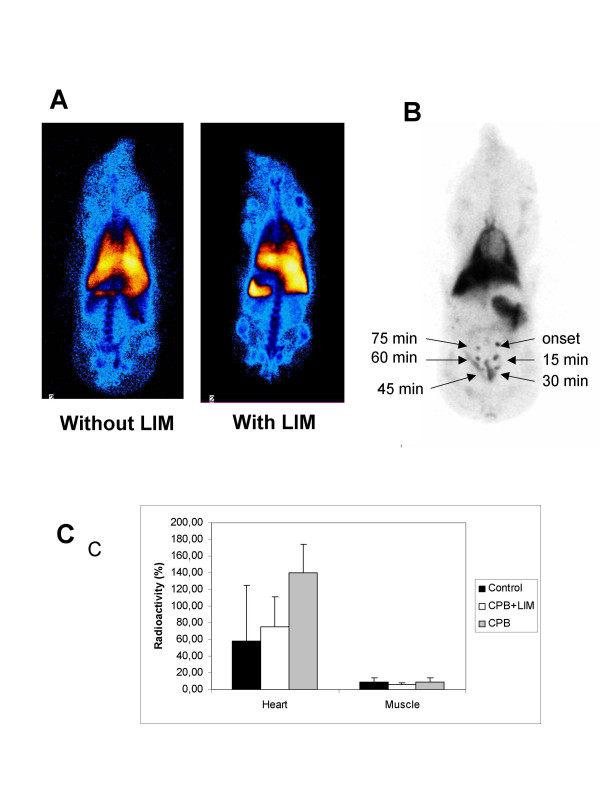
Whole body scintigraphy pictures from an animal without LIM or with LIM following injection of HMPAO-labeled neutrophils (A). High radioactivity was found in the spleen of LIM-treated animals. An internal control with subcutanously injected E.coli (control pig with CPB) confirmed the neutrophil activity over time (B). Data for the accumulation of radioactivity in the myocardium and musle tissue of control and LIM-treated animals is shown (C) as mean ± SD (CPB: n = 7; CPB + LIM: n = 8).

In order to determine the global distribution of neutrophils within the body after passing the LIM, technetium-labeled neutrophils were injected into the blood circulation before onset of CPB or CPB with LIM (n = 2, each group). One hour after end of surgery the distribution of the labeled neutrophils was analyzed by scintigraphy (Figure 6). In Figure 6A an example for the total body distribution of radioactivity is provided. In contrast to the control animal the depicted scintigraphy of the LIM-treated animal revealed no or only little radioactive load in heart and lung, whereas the spleen was significantly loaded. As an internal control, attenuated E.coli were injected subcutaneously at six different intraoperative time points (onset of CPB and subsequently each 15 minutes) to provoke neutrophil migration to the injection site (Figure 6B). Black spots indicate that labeled neutrophils retained their ability to infiltrate the challenged tissues throughout the entire operation time. Radioactivity determined in biopsies from heart and muscle (reference tissue) revealed that LIM prevented CPB-mediated accumulation of labeled neutrophils in the heart (2.69 × 10^6 ^± 1.19 and 4.30 ± 1.87 × 10^6^/g, respectively). Data is shown in percent of the applied radioactivity (Figure 6C) as the mean ± SD (CPB: n = 7; CPB + LIM: n = 8).

## Discussion

Recently, it has been reported that CPB impairs left ventricular contractility and cardiac function [25,26]. Herein, we showed that LIM when incorporated into the arterial line of the CPB system effectively stabilized perioperative cardiac function during CPB in the porcine model.

The pathophysiologic mechanisms underlying CPB-related impairment of cardiac function are not exactly known. However, it has been suggested that neutrophil activation that occurs during cardiac surgery using CPB may be strongly related with cardiac and pulmonary tissue damage after opening of the aortic cross clamp [7]. Following reperfusion of the ischemic heart and lung, hyperactivated neutrophils reach the capillaries of the pre-damaged tissues where further endothelial leakage and extracellular matrix destruction may occur due to neutrophil adhesion and transendothelial migration [27,28]. The local accumulation of chemokines and proinflammatory cytokines such as TNF-α further attracts and activates neutrophils that potentially degrade tissue integrity via oxygen radicals and proteases. Recently, we were able to show that neutrophil-mediated disruption of microvascular endothelial cell integrity correlates with prolonged CPB time [23]. For example, TNF-α seems to catalyze neutrophil-mediated tissue damage [29] and has been suspected to directly disturb pulmonary [30] and cardiac function [31].

From this knowledge it is conceivable, that perioperative prevention of neutrophil hyperactivity and inflammation may be an important tool to stabilize pulmonary and cardiac functionality that would result in better patient outcome. Therapeutic approaches with immunomodulating drugs or with leukocyte filtration have not been sufficiently effective to limit perioperative neutrophil activity in the past [16,17]. In some studies, leukocyte filtration rather activated proinflammatory responses probably due to the failure to rapidly inactivate stimulated neutrophils [32]. It has recently been shown that LIM immediately inhibits neutrophil function in an experimental porcine CPB model [18]. We therefore speculated that LIM might have also beneficial effects on the cardiac outcome following CPB.

A feasibility study with cardiac surgery patients already showed the proof of concept for LIM [24]. In this recent study LIM significantly prevented the perioperative increase in leukocyte numbers, neutrophil elastase, and TNF-α. These elements are known to contribute to the development of SIRS [33] and epithelial barrier dysfunction [34]. Moreover, CK and CK-MB values as indicators for tissue damage and myocardial injury, respectively were reduced with LIM compared with CPB without LIM [18]. However, the mechanisms by which LIM may protect heart and lung were unresolved.

From the herein presented data, we conclude that neutrophils may affect pulmonary and cardiac function during CPB and thus entail impairment of left ventricular contractility and increased pulmonary vascular resistance, both important features of cardiac function. The ESPVR and EDPVR values as markers for pre-load independent contractility and elastance of the left ventricle were significantly stabilized by LIM. Left ventricular outflow tract accelerated (LVOTacc) velocity, an additional pre-load independent contractility parameter measured by echocardiography [35], confirmed the beneficial effects of LIM (data not shown). The numbers of neutrophils that infiltrated the cardiac tissue upon CPB were relatively low. However, the numbers of infiltrated neutrophils were even lower in the LIM group. In contrast, the lung was drastically infiltrated by neutrophils after CPB but to a lesser extent in the LIM group. Although the possibility that the low number of neutrophils within the heart muscle may directly disturb the contractility of the left ventricle is unlikely, it has been shown that high levels of cardiac troponine I, MPO, and neutrophil numbers within the cardiac sinus are related to ischemia/reperfusion damage [36]. Moreover, it is rather likely that the neutrophil infiltration of the pulmonary tissue during CPB significantly increases the pulmonary vascular resistance (no-reflow phenomenon) [8-15] that in turn may affect the preload of the left ventricle.

Our preliminary findings obtained by scintigraphy support our assumption that LIM rapidly prevents hyperactivation of neutrophils and that preapoptotic neutrophils are effectively recognized by the immune system [20] and subsequently sequestered by the spleen.

## Conclusion

In our porcine model LIM proved to be an effective tool to limit neutrophil hyperactivation and prevent CPB-associated impairment of cardiac function. However, the link between organ neutrophil sequestration and cardiac function needs to be interpreted in caution, as both the morphological and scintigraphic data were obtained from a very limited number of animals.

An ongoing clinical study with LIM in patients undergoing cardiopulmonary bypass should confirm clinical efficacy and safety.

## Competing interests

SM partly works as a freelancer at Leukocare AG.

MS is CSO at Leukocare AG

The other authors declare that they have no competing interest.

## Authors' contributions

UA-R, JB-M and TA were responsible for the surgical procedures. IF and TL were responsible for the histological analyses and electron microscopy. SM, TK, and FG were responsible for the concept and logistics, as well as for the neutrophil labeling and measurement of radioactivity. JW, AM, and MS conceived of the study and were involved in drafting the manuscript. All authors read and approved the final manuscript.
